# The silencing of the SWI/SNF subunit and anticancer gene BRM in Rhabdoid tumors

**DOI:** 10.18632/oncotarget.1945

**Published:** 2014-05-04

**Authors:** Bhaskar Kahali, Jinlong Yu, Stefanie B. Marquez, Kenneth. W. Thompson, Shermi Y. Liang, Li Lu, David Reisman

**Affiliations:** ^1^ Division of Hematology/Oncology, Department of Medicine, University of Florida, Florida, USA; ^2^ Department of Pathology, University of Florida, Florida, USA

**Keywords:** SWI/SNF, Brahma-Related Gene 1, chromatin remodeling, Brahma, Rhabdoid

## Abstract

Rhabdoid sarcomas are highly malignant tumors that usually occur in young children. A key to the genesis of this tumor is the mutational loss of the BAF47 gene as well as the widespread epigenetic suppression of other key anticancer genes. The BRM gene is one such epigenetically silenced gene in Rhabdoid tumors. This gene codes for an ATPase catalytic subunit that shifts histones and opens the chromatin. We show that BRM is an epigenetically silenced gene in 10/11 Rhabdoid cell lines and in 70% of Rhabdoid tumors. Moreover, BRM can be induced by BAF47 re-expression and by Flavopiridol. By selective shRNAi knockdown of BRM, we show that BRM re-expression is necessary for growth inhibition by BAF47 re-expression or Flavopiridol application. Similar to lung cancer cell lines, we found that HDAC3, HDAC9, MEF2D and GATA3 controlled BRM silencing and that HDAC9 was overexpressed in Rhabdoid cancer cell lines. In primary BRM-deficient Rhabdoid tumors, HDAC9 was also found to be highly overexpressed. Two insertional BRM promoter polymorphisms contribute to BRM silencing, but only the -1321 polymorphism correlated with BRM silencing in Rhabdoid cell lines. To determine how these polymorphisms were tied to BRM silencing, we conducted ChIP assays and found that both HDAC9 and MEF2D bound to the BRM promoter at or near these polymorphic sites. Using BRM promoter swap experiments, we indirectly showed that both HDAC9 and MEF2D bound to these polymorphic sites. Together, these data show that the mechanism of BRM silencing contributes to the pathogenesis of Rhabdoid tumors and appears to be conserved among tumor types.

## INTRODUCTION

Rhabdoid sarcomas are rare, lethal pediatric sarcomas characterized by a 22q11 chromosome rearrangement that targets and inactivates the BAF47 (INI1, smarcb1) gene. BAF47 has been shown to be inactivated in the vast majority of these tumors via mutations, gene deletions, or both. However, in at least 10% of cases, this gene may be silenced through as yet unidentified mechanisms [1]. Heterozygous knockouts of BAF47 yield tumors in 30% of mice, a percentage that has been confirmed by 3 different groups [2]. Homozygous knockouts of BAF47, in comparison, resulted in tumor development in 100% of the mice (80% lymphomas and 20% Rhabdoid tumors) in a median time of 10-12 weeks [3]. *In vitro* studies have shown that re-expression of BAF47 in BAF47-deficient cell lines yields growth inhibition [4]. Together, these data solidified BAF47's role as a tumor suppressor that underlies the genesis of Rhabdoid tumors. While BAF47 silencing is considered very tumorigenic, this gene has not been found to be silenced in common adult tumors such as lung, prostate, esophageal, colon, or breast cancer [2]. Rather, BAF47 is silenced in less common tumor types, such as renal medullary carcinomas, epithelioid sarcomas, a subset of epithelioid malignant peripheral nerve sheath tumors, some lymphomas, myoepithelial carcinomas, and chondrosarcomas [5-7]. Interestingly, unlike lung, breast, and colon cancers, which harbor a variety of mutations and alterations, NextGen sequencing has indicated that Rhabdoid tumors have a low number of mutated genes (~10); instead, it is surmised that a large number of epigenetic changes drive the progression of this lethal tumor type [8]. The investigation of these epigenetically silenced genes in Rhabdoid tumors is required to gain a better understanding of why this tumor type is so lethal.

Here, we explore and define the role of epigenetic silencing of the anticancer gene, Brahma (BRM), in Rhabdoid cancer. This gene, like BAF47, is one of approximately 9 subunits that assemble to form the SWI/SNF chromatin remodeling complex [9, 10]. This complex has a generic role in gene expression, as it is recruited by key cellular proteins and transcription factors to shift the position of histones within chromatin, thereby opening up the DNA and facilitating gene expression [11-13]. This complex's actions are tied to a plethora of cellular functions that oppose cancer development, including growth control, DNA repair, cellular adhesion, differentiation, and development. [2, 14]. Disruption or inhibition of the SWI/SNF complex through the loss of one or more of the subunits negatively impacts these cellular processes, and it is therefore not surprising that the loss of SWI/SNF function potentiates cancer development. BRG1 and BRM silencing are important for cancer development, as their function is a prerequisite for the function of a number of anticancer tumor suppressor proteins such as p53, BRCA1 and Rb. In fact, BRM and BRG1 both bind to the Rb protein and are required for Rb-mediated growth inhibition; functional loss of BRM, BRG1, or both *in vitro* blocks and/or abrogates Rb function. Similarly, the loss of BRM and BRG1 function can inactivate the Rb homologs p107 and p130, which control G2/S phase progression and the transition from G1 to G0, respectively.

Importantly, like the impact of the loss of BRG1 and BRM on the Rb pathway, BAF47 loss also appears to affect the Rb pathway. Loss of BAF47 in Rhabdoid cell lines correlates with over expression of EZH2, an oncogenic methyltransferase involved in gene silencing. This induction of EZH2 in turn epigenetically leads to the silencing of p16, which then leads to the phosphorylation and inactivation of Rb [15]. Although Rb is made functional by the induction of p16, Rb still requires BRG1 and/or BRM to foster Rb-mediated growth inhibition. Re-expression of BAF47 induces growth arrest by driving the dephosphorylation of Rb by this mechanism. This observation is important because it shows that different SWI/SNF subunits possess different roles as part of a common mechanism: Rb pathway activation. The observation that HDAC inhibitors can reverse BRM silencing [16, 17] indicates that the restoration of BRM could represent a novel form of targeted therapy. This concept is supported by the fact that restoring BRM causes growth inhibition and differentiation to occur [2]. These observations led us to pursue the identification of potential agents that could restore BRM, and using high throughput screening with a functional BRM assay, we found a number of different compounds capable of restoring BRM function [18]. By screening libraries of natural products and FDA-approved compounds, we found a relatively high number of compound hits were from the same family, namely flavonoids. Further studies have shown that essentially each compound tested from this family could readily induce BRM and resulted in BRM-dependent growth inhibition [19]. Based on these data and the fact that the synthetic flavonoid Flavopiridol inhibits growth in Rhabdoid tumors [20], we investigated the extent to which BRM silencing might be involved in Rhabdoid tumors and observed that BRM was silenced in the majority of Rhabdoid cell lines (10/11) and in ~65-70% of primary Rhabdoid tumors. Moreover, we have found that the mechanism of action of Flavopiridol involves the reactivation/induction of BRM as a means to restore Rb-mediated growth inhibition in addition to its ability to inhibit cdk2/4, yielding an activated Rb.

We previously found that, central to BRM silencing, is the presence of two germline insertional 6 base pair polymorphisms in the promoter of BRM [21]. These polymorphisms statistically correlate with BRM loss both in cancer cell lines and in primary lung cancers [21]. While BRM loss in mice is not by itself tumorigenic, BRM loss does potentiate cancer development when combined with carcinogens [17]. This suggests that BRM is not a tumor suppressor gene but rather a gene that can facilitate cancer development: that is, a tumor susceptibility gene. Since these BRM polymorphisms correlate with BRM loss [21], and BRM loss potentiates cancer development [17], we surmised that these BRM polymorphisms might be predictive of cancer development. To this end, we have shown that these BRM polymorphisms, and indirectly BRM loss, are statistically correlated with cancer risk with an odds ratio of 2.4-3.0 [21, 22]. In addition, these polymorphisms and BRM loss are known to predict poor clinical outcomes in lung cancer [23, 24]. In this paper, we show that in Rhabdoid cell lines, the -1341 polymorphism correlates with the loss of BRM. Similar to our published studies with lung cancer, we found that BRM was also regulated by HDAC3, HDAC9, GATA3, and MEF2D in Rhabdoid tumors [25]. We also observed that HDAC9 was overexpressed [25] in Rhabdoid cancer cells as well as in lung cancer cells. Since these polymorphisms have a homology of about >90% with MEF2 binding sites, we surmised that these BRM polymorphisms are targeted and bound by MEF2D and HDAC9, since HDAC9 has been shown to be recruited by MEF2D [26]. Using Chromatin Immunoprecipitation (ChIP) experiments, we found that in Rhabdoid cell lines, MEF2D and HDAC9 were bound to the BRM promoter when these polymorphisms were present but did not bind in their absence. Hence, the epigenetic mechanism of BRM silencing appears to be conserved among tumor types, given that it appears to be similar if not identical in lung cancer and Rhabdoid tumors. Moreover, we found that BAF47 regulated BRM and that BAF47-mediated growth inhibition was BRM-dependent, functionally tying BRM to BAF47 as part of the mechanism that underlies the genesis of Rhabdoid tumors.

## RESULTS

### BRM Loss in Rhabdoid Cell Lines

**Figure 1:**
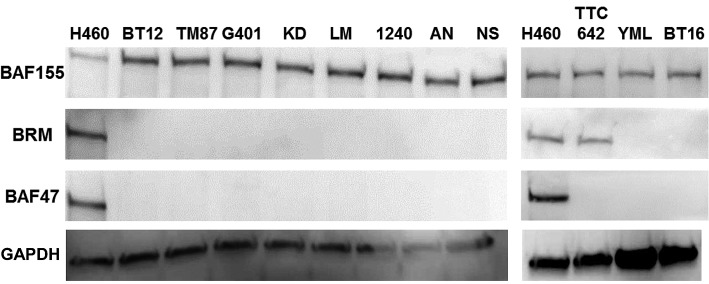
In a western blot analysis, 11 Rhabdoid cell lines were probed for BRM, BAF47 and BAF155 expression where H460 was used as the positive control These Rhabdoid cell lines have been observed to be BAF47-negative. Ten of 11 other Rhabdoid cell lines were BRM- negative, and TTC-642 was the only Rhabdoid cell line found to be BRM-positive. As BAF155 is sensitive to degradation, the presence of BAF155 expression in all cell lines including the positive control, H460, demonstrates that degradation in these samples is not likely to have occurred. GAPDH was used as the loading control.

The synthetic flavonoid, Flavopiridol, has been shown to robustly inhibit the growth of Rhabdoid cell lines [20]. Since we previously found by high throughput screening that a number of flavonoids can induce BRM and activate BRM via deacetylation [18], we suspected that BRM re-expression could contribute to the growth inhibition that was induced by Flavopiridol treatment in Rhabdoid cell lines. We obtained 11 Rhabdoid cell lines and conducted western blotting for the absence of BAF47; we also sequenced p53 in these cell lines for the absence of mutations to establish that they are consistent with a Rhabdoid tumor phenotype. Each of the 11 Rhabdoid cell lines lacked BAF47 expression (Figure 1) and lacked any detectible expression, as predicted by western blot (data not shown) as well as any p53 mutations as evidenced by Sanger sequencing. We then genotyped each cell line for the presence of the BRM polymorphisms (Supplementary Table 1) [21]. Of these 11 cell lines, 10 demonstrated genetically distinct molecular profiles for BRM polymorphism patterns, p53 mutations and BAF47 mutations. By sequencing genomic DNA for the 9 exons of BAF47, we found that 6 out of the 11 cell lines harbored the same BAF47 mutations as previously described [27, 28], whereas the mutation status of the other 5 cell lines had not yet been published. We observed that BAF47 was deleted in 3 of these cell lines (KPMRT-AN, BT12 and BT16), while the other 2 cell lines, KPMRT-YML and KPMRT-NS, were devoid of detectible mutations (Supplementary table 1). Of the 11 cell lines, the BAF47 changes were distinctly different in 10 (Supplementary table1). Thus, at least 10 of the 11 cell lines were unique Rhabdoid cell lines. Two of these 11 cell lines (BT16 and G401) had the same genotypic pattern (BRM polymorphisms and BAF47 deletion) but were obtained from completely different sources, and thus, we considered them to be unique. As such, we proceeded to analyze 11 cell lines.

We conducted western blotting for BRM in each of these 11 Rhabdoid cell lines and observed that 10 out of 11 lines were completely devoid of BRM expression (Figure 1); only the TTC-642 cell line was found to be BRM-positive and had levels of BRM expression comparable to the positive control cell line H460. This finding was consistent with data from Muchardt and Yaniv in their review paper [29], where they reported (as unpublished data) that at least 5/5 cell lines (Wa2, KD, LP, DL, and G401) were BRM-deficient. Hence, together with the data that was reported by Muchardt *et al*.[29] at least 13/14 Rhabdoid cell lines have been reported to be deficient for BRM expression. As the SWI/SNF subunit BAF155 is sensitive to protein degradation (personal communication, Bernard Weissman), we also examined BAF155 via western blotting to rule out the possibility of degradation. To this end, we observed that BAF155 was robustly expressed in each of these 11 cell lines (Figure 1) thereby showing that degradation was not likely occurring in these protein samples.

### BRM Loss in Primary Rhabdoid Tumors

**Figure 2:**
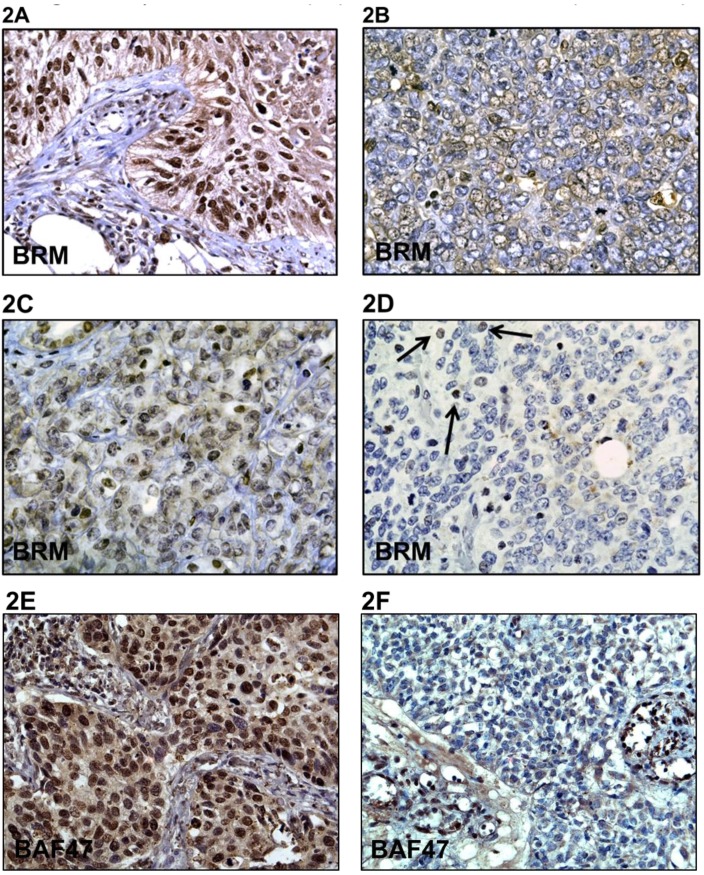
A-F demonstrates immunohistochemical (IHC) staining for BRM in Rhabdoid tumors and in a positive control lung cancer as well as staining for BAF47 in Rhabdoid tumors **A** is the positive control that shows BRM immunoreactivity in a non-small cell lung tumor. **B** and **C** demonstrate low to moderate BRM staining, respectively. **D** illustrates the lack of BRM staining in Rhabdoid tumors, where the black arrows indicate internal positive controls. **E** illustrates positive BAF47 staining in a non-small cell lung tumor (positive control), whereas **F** illustrates the absence of BAF47 expression in Rhabdoid tumors by IHC.

As cell lines do not always recapitulate the genetic changes that occur in primary tumors, we analyzed the expression of BRM in 29 paraffin-embedded primary Rhabdoid tumors. For these experiments, we used a BRM polyclonal double-immunopurified antibody, which we have shown in previous publications to be both sensitive and specific to BRM expression and which does not cross-react with other proteins such as BRG1 [17]. Immunohistochemical (IHC) staining of these 29 histologically verified Rhabdoid tumors with this polyclonal BRM antibody revealed that these tumors were either devoid of BRM expression (~62%) or had very low levels of BRM expression (28%) (Figure 2D), while 10% had moderate staining (Figure 2B & 2C; Table 1). However, no Rhabdoid tumors were observed to have intense BRM staining, which is typically observed in about 30% of non-small cell lung cancers (Figure 2A) [30]. In addition, we found that these tumors were devoid of BAF47 expression by IHC, consistent with the Rhabdoid phenotype (Figure 2F). Given the fact that Rhabdoid tumors are nearly always devoid of BAF47 expression, non-small cell lung tumors were used as the positive control for BAF47 expression in IHC (Figure 2E). Together, these data indicate that BRM loss is a consistent molecular change that occurs in Rhabdoid tumors.

Table 1An immunohistochemical analysis was performed for all 29 Rhabdoid tumorsSections were scored according to staining intensity (0, +1, +2, +3) and percentage of tumor cells stained (1-100%). The product of these two values was then obtained for each tumor. In the table 1A, the tumor designation is listed in the first column, and the source of each tumor is listed in the second column. The percentage, intensity, and product for each specimen are then given in the next 3 columns, respectively. The intensity is given as an average of the intensities of at least 4 distinct areas within each tumor section. Tumors with a staining product of 25 or less were deemed negative for BRM; as some investigators set a cut-off as high as 50 for negative samples, our calculation of 62% of tumors that are negative for BRM expression is a relatively conservative one. If we had used a cut-off value of 50, then 80% of our tumor specimens would have been deemed negative for BRM expression. COG=Children's Oncology Group; UF=University of Florida; UM=University of Michigan. In the 1B, the number and percentage of tumors with a specific range of product values are given, as is the classification of the tumor as negative, low, moderate or high with respect to BRM immunoreactivity.

**1A:** 

Tumor	Source	%	Intensity	Product
PASDLA-OAAG7O	COG	0.0%	0.00	0.00
PATJSF-OAAGRG -	COG	1.3%	0.25	0.31
PATJJF-OAAGRD	COG	1.3%	0.25	0.31
PAUCGJ-OAAGT3	COG	1.3%	0.50	0.63
PARECB-OAEM1P	COG	1.3%	2.00	2.50
S03-3138C12	UF	1.3%	2.00	2.50
PASILR-OAAG78	COG	12.5%	0.25	3.13
PARIRN-OAEM1T	COG	5.0%	0.75	3.75
PAUHAZ-OAAGTZ	COG	8.8%	0.50	4.38
UM-3	UM	5.0%	1.25	6.25
UM-1	UM	5.5%	1.75	9.63
PASNED-OAEM21	COG	10.0%	1.00	10.00
PARUGK-OAAG5O	COG	15.0%	0.75	11.25
PAUEKW-OAEPZA	COG	6.3%	2.00	12.50
UM-2	UM	12.5%	1.00	12.50
S03-21049A6	COG	15.0%	1.00	15.00
PASRHU-OAAG78	COG	10.0%	1.50	15.00
PATNBW-OAEM2E	COG	10.0%	1.50	15.00
PASXNA-OAEM29	COG	27.5%	1.00	27.50
S98-11427A1	UF	27.5%	1.00	27.50
UM-4	UM	20.0%	1.67	33.32
PATUXL-OAAGRH	COG	42.5%	1.00	42.50
PATENH-OAAGWJ	COG	32.5%	1.50	48.75
PASXGF-OAEM25	COG	33.8%	2.00	67.50
S02-01130A1	UF	53.8%	1.28	68.90
S05-8116B2	UF	61.3%	1.25	76.56
PARPFY-OAEM1X	COG	57.5%	2.25	129.38
PATFXW-OAAGWJ	COG	87.5%	1.50	131.25
PATFXW-OAAGQD	COG	87.5%	1.50	131.25

**1B:** 

Product	No#	%	Catalog
0-25	18	62%	Negative
25-80	8.0	28%	Low
80-200	3.0	10%	Moderate
200-300	0.0	0%	High
Total	29		

### Flavonoids Reverse BRM Silencing

**Figure 3:**
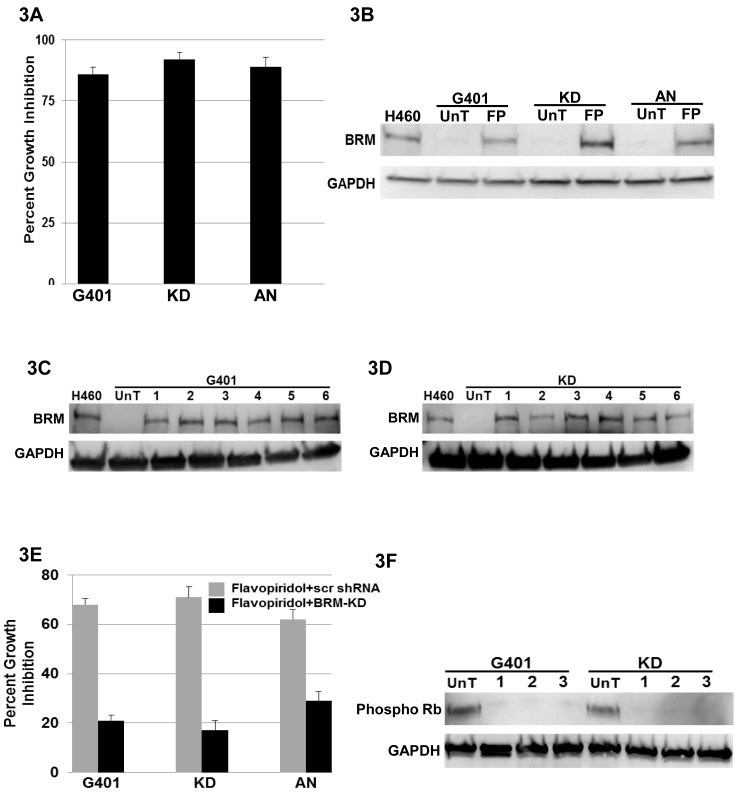
A demonstrates cellular growth inhibition (80-90%) following the transfection of BRM in the Rhabdoid cell lines G401, KD and KPMRT-AN over a period of 5 days **B** illustrates the induction of BRM protein in Rhabdoid cell lines, G401, KD and KPMRT-AN, following treatment with 250nM of Flavopiridol for 72 hours. “UnT” denotes the untreated parental cell line, and “FP” denotes the Flavopiridol-treated cell lines. H460 was used as the positive control, and GAPDH was used as the loading control. **C** and **D** demonstrate the induction of BRM protein in G401and KD cell lines, respectively, following a 72-hour treatment with 3µM flavonoids from each of the six flavonoid structural groups [1: Luteolin (flavone), 2: Quercetin (flavonol), 3: Genistein (isoflavone), 4: Hespiridin (flavanone), 5: EGCG (flavanol), and 6: Delphinidin (anthocyanin)]. UnT denotes the untreated parental cell line, and H460 was used as the positive control. GAPDH was used as the loading control. **E** The Rhabdoid cell lines, G401, KD and KPMRT-AN, harboring either scrambled shRNA (grey bar), or anti-BRM shRNA (black bar), were treated with 250nM of Flavopiridol. Daughter cell lines harboring the scrambled shRNA elicited considerable growth inhibition (65-70%) over 5 days following the treatment with Flavopiridol. In comparison, growth inhibition was significantly attenuated (20-30%) in cell lines harboring the anti-BRM shRNA (p<0.05). **F** demonstrates the reduction in the level of phospho-Rb in the G401 and KD cell lines following the treatment with (1) 250nM of Flavopiridol, (2) 3µM Luteolin, and (3) 3µM Quercetin for 72 hours. “UnT” denotes the untreated parental cell lines. GAPDH was used as the loading control.

Our lab and others have previously shown that transient transfection or viral infection of BRM/BRG1-deficient cell lines with BRM causes growth inhibition [17, 31]. To confirm this in Rhabdoid cell lines, we transduced 4 Rhabdoid cell lines with BRM and showed that each cell line was significantly growth-inhibited (Figure 3A). As published data has demonstrated that Rhabdoid tumors can be inhibited by Flavopiridol [20], we surmised that Flavopiridol might induce BRM. We therefore tested the effects of Flavopiridol on 3 BRM-deficient Rhabdoid cell lines (G401, KD, and KPMRT-AN) and found that BRM mRNA was induced at least 8-fold by 250nM of Flavopiridol as measured by qPCR (Supplementary Figure 1); similarly, by western blot, we observed that BRM protein was readily induced by 250nM Flavopiridol (Figure 3B). As such, Flavopiridol is one of the most potent inducers of BRM that we have observed to date. To determine if other flavonoids could induce BRM in a similar fashion, we tested one flavonoid from each of the six known structural groups. We treated two Rhabdoid cell lines (G401 and KD) with ~3µM of each of these flavonoids, and we observed by western blot that BRM protein was induced by the representative flavonoid from each structural group (Figure 3C and 3D), respectively. These data suggest, in general, that flavonoids can re-activate BRM. We previously found that flavonoids induce BRM in non-Rhabdoid cell lines in part by causing a significant down-regulation of HDAC9 (>70%); consistent with this data, we found that flavonoids also down-regulate HDAC9 in Rhabdoid cell lines (Supplementary Figure 2B). Given that BRM can be induced by MAPK inhibitors [25], it is not surprising that these compounds can induce BRM, as flavonoids including Flavopiridol have been found to be MAPK inhibitors [32, 33].

### BRM is Required for Flavonoid-Mediated Growth Inhibition

We also observed that Flavopiridol, along with each of the other tested flavonoids, induced growth inhibition. As BRM re-expression inhibits growth, we predicted that BRM induction may be involved in the mechanism of flavonoid-mediated growth inhibition in Rhabdoid cell lines. We tested Flavopiridol, Luteolin or Quercetin in 3 Rhabdoid cell lines (G401, KD, and KPMRT-AN) that were transduced with either scrambled or antiBRM shRNA. In each cell line, we observed robust growth inhibition in the cell lines transduced with scrambled shRNA (65-70%); however, this growth inhibition was blunted in the cell lines harboring antiBRM shRNA (15-25%; Figure 3E and Supplementary Figure 2). This finding is congruent with past publications where BRM has been shown to cooperate with Rb in order to inhibit cell growth [2, 34]. To this end, some flavonoids are known to inhibit CDKs which results in hypophosphorylated Rb [35-37]. To show the impact of flavonoids on Rb phosphorylation in Rhabdoid cell lines, we conducted western blotting on the KD and G401 cell lines and found that, indeed, the application of Flavopiridol, as well as of Luteolin and Quercetin, not only induced BRM but also decreased the levels of hyper-phosphorylated RB (Figure 3F).

### Mechanism of BRM Loss in Rhabdoid Cell Lines

**Figure 4:**
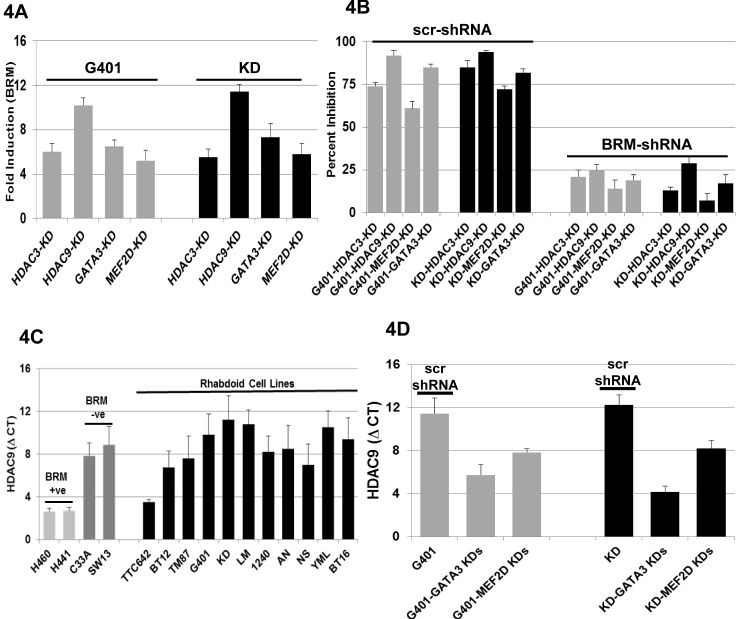
A shows the induction of BRM mRNA following the gene-specific shRNA-mediated knockdown of HDAC3, HDAC9, GATA3 or MEF2D in G401 and KD cell lines, which resulted in a greater than > 5-fold induction for each gene in either cell line B shows the G401 and KD cell lines harboring either scrambled shRNA (scr-shRNA) or anti-BRM shRNA; these cell lines were then subjected to gene-specific shRNA-mediated knockdown of HDAC3, HDAC9, MEF2D or GATA3. The knockdown of HDAC3, HDAC9, MEF2D or GATA3 displayed a statistically significant degree of growth inhibition in the cell lines harboring the scrambled shRNA (65-80%) in comparison to the daughter cell lines harboring the antiBRM shRNA (15-30%; p<0.05). C illustrates the level of HDAC9 mRNA in 11 Rhabdoid cell lines. Four previously characterized non-Rhabdoid cell lines, H441, H460 (BRM-positive:low HDAC9), and SW13 and C33A (BRM-negative: high HDAC9) were used as negative and positive controls, respectively, for HDAC9. The level of HDAC9 mRNA is approximately the same as in the BRM-negative non-Rhabdoid cell lines, SW13 and C33A, and on average is ~47±13 fold higher than the HDAC9 mRNA level observed in the BRM-positive Rhabdoid cell line, TTC642. D demonstrates the change in HDAC9 mRNA level following the gene-specific shRNA-mediated knockdown of either GATA3 or MEF2D. MEF2D knockdowns caused 15- and 16-fold down-regulation of HDAC9 in G401 and KD cells, respectively, compared to the cells harboring the scr-shRNA. GATA3 knockdowns resulted in a 75- and 256-fold down-regulation of HDAC9 in G401 and KD cells, respectively.

We previously found that the transcription factors MEF2D and GATA3, as well as the histone deacetylases HDAC3 and HDAC9, regulate BRM expression in BRM-deficient cancer cell lines [25]. As these proteins are known to form complexes with one another [26, 38], these results suggest that a complex of proteins regulates BRM. As a first step, we sought to determine if the mechanism of BRM regulation was the same or different in Rhabdoid tumor cells as compared to 2 previously studied BRM-deficient cancer cell lines, SW13 and C33A [25]. To accomplish this, we selectively knocked down the expression of HDAC9, HDAC3, MEF2D, and GATA3 using shRNA approaches. We observed that these gene knockdowns induced BRM mRNA 6-11-fold in the G401 and KD Rhabdoid cell lines (Figure 4A). We also observed that the suppression of these genes inhibited cell growth (65-80%) over a 5-day period (Figure 4B). To determine if the observed growth inhibition was functionally tied to BRM, we infected Rhabdoid cell lines with either antiBRM shRNA or scrambled shRNA (control). When each gene was selectively knocked down, we observed growth inhibition in the control cell lines harboring the scrambled shRNA. In contrast, we observed blunted growth inhibition (15-30%) in the Rhabdoid cell lines harboring antiBRM shRNA as compared to the control cell lines harboring scrambled shRNA, which demonstrated 65-85% growth inhibition (Figure 4B).

Previously, we found that changes in HDAC9 protein expression parallel the changes observed in HDAC9 mRNA levels [25]. Hence, we measured the change of HDAC9 expression by measuring HDAC9 mRNA levels by qPCR. Similar to our findings in other BRM-deficient cancer cells lines and primary lung cancers [25], we found that HDAC9 mRNA was overexpressed ~47±13-fold in Rhabdoid cell lines as measured by qPCR (Figure 4C). After the knockdown of MEF2D, we observed a reduction in HDAC9 mRNA expression by 15- and 16-fold in both the G401 and KD cell lines, respectively (Figure 4D). Similarly, the knockdown of GATA3 resulted in the reduction of HDAC9 mRNA by 75- and 256-fold in G401 and KD cell lines, respectively (Figure 4D). These findings suggest that overexpression of HDAC9 mRNA is due in part to the transcriptional activity of GATA3 and MEF2D, which is not surprising since both of these transcription factors are known to bind to the HDAC9 promoter [39]. Knockdown of HDAC3 had no impact on HDAC9 expression (Supplementary Figure 3), but readily induced BRM and caused BRM-dependent growth inhibition (Figure 4A and Figure 4B), which paralleled our observations in the non-Rhabdoid BRM-deficient cancer cell lines SW13 and C33A [25].

**Figure 5:**
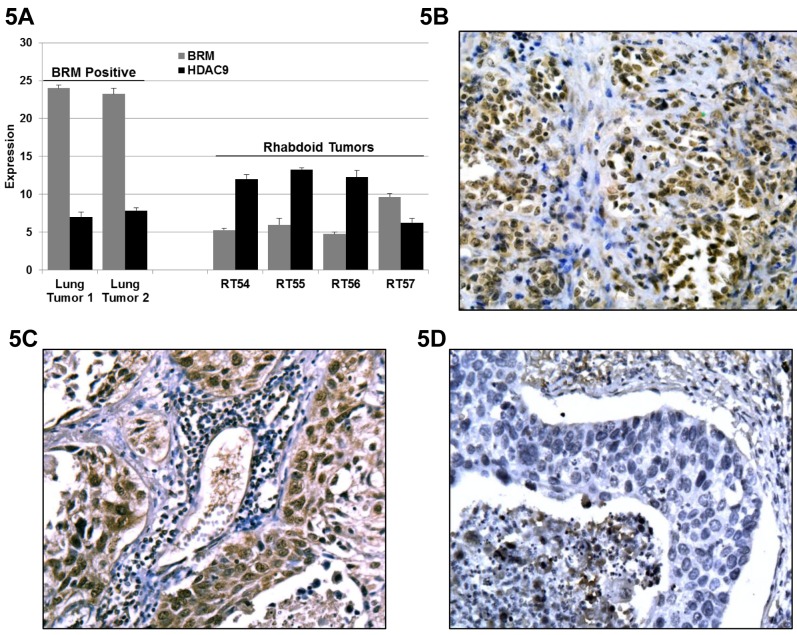
A Three BRM-negatives, 1 BRM-positive Rhabdoid and 2 BRM-positive lung tumors (positive controls) were analyzed for HDAC9 expression by qPCR The level of BRM mRNA between the BRM-positive (lung cancer) tumors and the BRM-negative (Rhabdoid) tumors is 2E18-fold higher (p< 0.0001). In addition, the level of BRM mRNA between the BRM low-moderate positive (Rhabdoid) tumor and the BRM-negative (Rhabdoid) tumors is 27±3 fold-higher (p< 0.01). The level of HDAC9 mRNA between the BRM-positive (lung cancer) tumors and the BRM-negative (Rhabdoid) tumors is ~22-fold lower (p< 0.03). In addition, the level of HDAC9 mRNA between the BRM low-moderate positive (Rhabdoid) tumor and the BRM-negative (Rhabdoid) tumors is ~90-fold lower (p< 0.01). Hence, there is an inverse correlation between BRM and HDAC9 mRNA expression levels in Rhabdoid tumors. Fold differences of HDAC9 mRNA expression were calculated by subtracting the average ΔCt value of HDCA9 mRNA (as measured by qPCR) in BRM-positive cancer cells from the average ΔCt value of HDCA9 mRNA in BRM-negative cancer cell lines and raised to the base “2”. Specifically, the formula is: 2^(averageΔCT of HDAC9 in BRM-negative cell lines – averageΔCT of HDAC9 in BRM-positive cell lines)^ = fold difference. B-D representative Rhabdoid and lung tumors, immunohistochemically stained with anti-HDAC9 antibody. B and C show the expression of HDAC9 in Rhabdoid (BRM-negative) and lung tumors (BRM-negative), respectively, as compared to D which shows almost no HDAC9 staining in the BRM-positive lung tumor.

We next examined the mRNA expression level in 3 BRM-deficient and 1 BRM-positive Rhabdoid tumors, as determined by IHC, and observed that the BRM mRNA expression was on average 27±3-fold lower in the BRM-negative Rhabdoid tumors compared to the BRM-positive tumor. In these same tumors we observed the inverse correlation with HDAC9 expression. Specifically, we observed that HDAC9 expression in 3 BRM-deficient Rhabdoid tumors was 80±25-fold higher as compared to the BRM-positive Rhabdoid tumors (Figure 5A). These observations were similar to our published findings where we observed that HDAC9 expression was 500- and 50-fold higher in BRM-deficient lung cancer cell lines and BRM-deficient primary lung tumors, respectively, compared to BRM-positive lung cancer cell lines and primary tumors [25]. We next immunostained for HDAC9 and found that HDAC9 was qualitatively overexpressed in Rhabdoid tumors (Figure 5B) compared to the HDAC9-positive non-small cell lung tumor (positive control: Figure 5C) and the HDAC9-negative (negative control: Figure 5D) lung tumor. These data demonstrate that HDAC9 is over expressed in BRM-deficient cancer cells. As the knockdown of HDAC9 induces BRM in both non-Rhabdoid [25] as well as in Rhabdoid cancer cell lines, these data support the hypothesis that HDAC9 is central to the epigenetic suppression of BRM in human tumors.

### Re-expression of BRM Inhibits Rhabdoid Cell Growth

**Figure 6:**
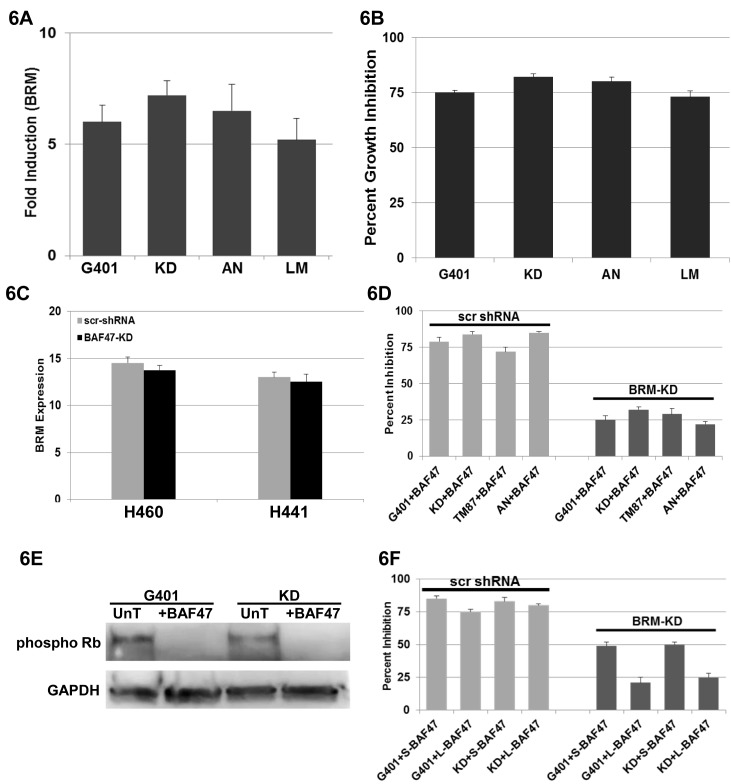
A illustrates the induction of BRM mRNA by 5-7-fold as measured by qPCR in 4 Rhabdoid cell lines, G401, KD, KPMRT-AN and LM, following transfection of BAF47 B demonstrates cellular growth inhibition (~80%) following the transfection of BAF47 in Rhabdoid cell lines, G401, KD, TM87 and KPMRT-AN, over a period of 5 days. C shows the level of BRM mRNA following the gene-specific shRNA-mediated knockdown of BAF47 in two BRM-positive, non-Rhabdoid cell lines, H441 and H460. No significant changes in BRM mRNA were observed between the daughter cell lines harboring the scrambled shRNA or the anti-BAF47 shRNA (p>0.05). D shows the G401, KD, TM87 and KPMRT-AN cell lines which harbor either scrambled shRNA (scr-shRNA) or anti-BRM shRNA and that were also transduced with BAF47. Daughter cell lines harboring the scrambled shRNA elicited growth inhibition (~70-80%) over a period of 5 days following the transfection of BAF47. In comparison, growth inhibition was significantly attenuated (~25-30%) in cell lines harboring the anti-BRM shRNA (p<0.05). E demonstrates the reduction in the phospho Rb level in G401 and KD cell lines following the transduction of BAF47. “UnT” denotes the untreated parental cell lines. GAPDH was used as the loading control. F G401 and KD cell lines harboring either scrambled shRNA (scr-shRNA) or anti-BRM shRNA were transduced with the short form (S-BAF47) or with the long form (L-BAF47) of BAF47. Daughter cell lines harboring the scrambled shRNA (scr-shRNA) elicited appreciable growth inhibition (~75-80%) over a period of 5 days following the transfection of either L-BAF47 or S-BAF47. The S-BAF47 transduced into the daughter cell line harboring antiBRM shRNA showed a greater degree of growth inhibition (~50%; p<0.05) than same cell line transduced with the L-BAF47 (~25%).

Re-expression of BAF47 induces growth inhibition by down-regulating EZH2, which in turn induces p16 [15]. While the induction of p16 is sufficient to activate Rb, our prior experiments suggested that BRM can foster growth inhibition in Rhabdoid cell lines. This would be expected, since we and others have found that BRM binds to Rb (and Rb2:p130) through its LXCXE region, and is a cofactor for Rb-mediated growth inhibition [30, 34, 40]. We therefore surmised that the re-expression of BAF47 might also induce BRM. To test this hypothesis, we transfected 4 Rhabdoid cell lines (G401, KD, KPMRT-AN, and LM) with a BAF47 expression vector, and using qPCR, we measured the changes in BRM expression. We observed that BAF47 re-expression induced BRM mRNA (~5-7-fold) (Figure 6A) as well as growth inhibition (~80%) (Figure 6B) over a period of 5 days. We similarly observed the induction of BRM protein after BAF47 transfection in these cell lines (data not shown). As HDAC9 overexpression is linked to BRM silencing, we investigated whetherBAF47 re-expression impacted HDAC9 expression. Unlike the impact of flavonoids, which induce BRM by down-regulating HDAC9, BAF47 re-expression had no appreciable impact on HDAC9 mRNA expression as measured by qPCR (Supplementary Figure 4B). We next tested whether the converse relationship could be observed: that is, if we knocked down BAF47 in a BRM-positive/BAF47-positive cell line, would we observe down-regulation of BRM expression? Since all Rhabdoid cell lines are BAF47-negative, we used the established ATCC lung cancer cell lines H460 and H441, which are positive for both BRM and BAF47, to further investigate BAF47 regulation of BRM. In the H460 and H441 lung cancer lines, we knocked down BAF47 using antiBAF47 shRNA approaches. As changes in BRM mRNA correlate with changes in BRM protein, we conducted qPCR to qualitatively measure the changes in BRM expression [17, 25]. After BAF47 knockdown in these two cell lines (Supplementary Figure 4A), we observed no significant change in the BRM mRNA levels (p>0.05) (Figure 6C). Hence, the functional relationship between BRM and BAF47 may be restricted to only Rhabdoid cancer cells. We next determined if this BAF47-induced growth inhibition required BRM re-expression. We repeated this experiment of BAF47 re-expression in Rhabdoid cell lines except that we transduced each cell line with either scrambled or anti-BRM shRNA. In the daughter Rhabdoid cell lines transduced with scrambled shRNA, we observed growth inhibition after BAF47 re-expression (~70-80%). In comparison, in the Rhabdoid cell lines harboring anti-BRM shRNA, we only observed ~25-30% growth inhibition after BAF47 re-expression (Figure 6D). In addition, we observed that Rb becomes dephosphorylated after BAF47 re-introduction into G401 and KD Rhabdoid cell lines (Figure 6E). Combined, these data show that both flavonoid treatment and BAF47 re-expression may facilitate growth inhibition in Rhabdoid cell lines by not only converting Rb into its hypophosphorylated form but also by inducing BRM.

Like many genes, the BAF47 gene is alternately spliced into two different isoforms. The longer BAF47 isoform has the addition of 27bp in exon 2 that the short BAF47 isoform lacks [41, 42]. As there may be differences in the functionality of these BAF47 splicing variants, we tested each of these isoforms (long and short). In triplicate experiments, we observed 85% and 83% growth inhibition with the short BAF47 isoform in KD and G401 cell lines, respectively, as compared with 75% and 80% with the long BAF47 isoform in these same cell lines (Figure 6F). Hence, we did not observe any statistically significant (p>0.05) difference in growth inhibition when each of these two isoforms was re-expressed. We also tested the impact of the BAF47 isoforms on growth inhibition if BRM induction was blocked by antiBRM shRNA. In this case, we observed 49% and 50% growth inhibition when using the short form as compared to 21% and 25% growth inhibition (N=3) when using the long form in the KD and G401 cell lines, respectively (Figure 6F). Thus, the short BAF47 isoform resulted in ~2-fold more growth inhibition (p<0.05) as compared to the longer BAF47 isoform. To determine if these results occurred because of different levels of residual BRM (different efficiencies of BRM knockdown), we conducted western blots for BRM in each of the BRM knockdown cell lines after the re-expression fo the BAF47 long and short isoforms. After re-expression with either the long or short form of BAF47 in the Rhabdoid cell lines harboring antiBRM shRNA, we did not observe any significant difference in the very low residual BRM expression levels as measured by densitometric analysis (using NIH Image J software) (Supplementary Figure 5). These data suggest that the long isoform of BAF47 may be more dependent on BRM induction based on its observed growth inhibition in these cell lines.

### HDAC9 and MEF2D Binding of the BRM Polymorphic Sites and the Inhibition of BRM Expression

**Figure 7:**
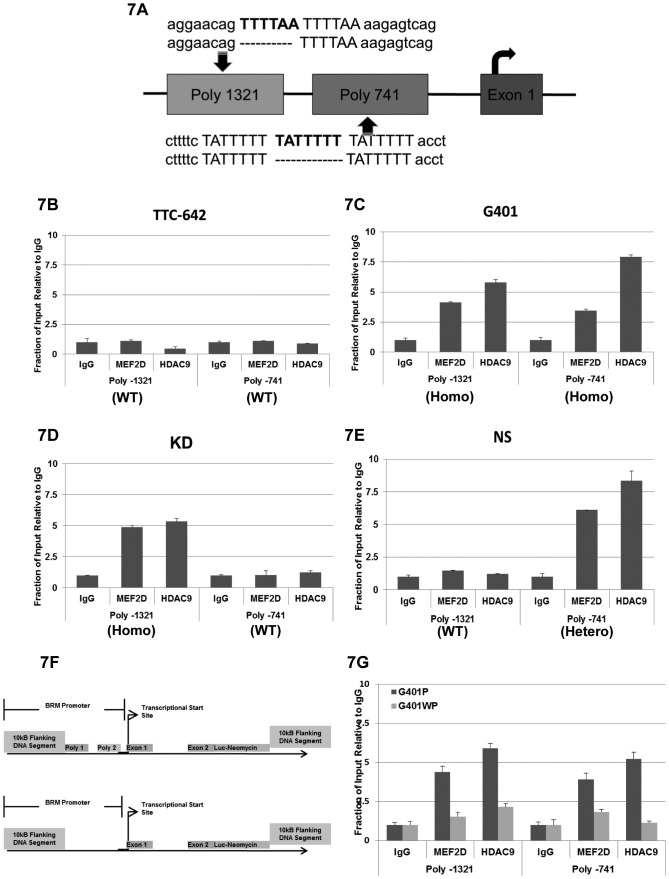
A illustrates the two BRM insertion polymorphisms (in bold), -1321 and -741, which are 1321 bp and 741 bp, respectively, upstream of the transcription start site The 1321 polymorphic site is a duplicate repeat of the “TTTTAA” sequence, whereas the -741 polymorphic site is a triplicate repeat of the “TATTTTT” sequence. The position of the first exon is shown as well as curved arrow, which designated the transcription start site. The wild type or nonpolymorphic sites are represented by the absence of the additional sequence (polymorphic site) by a broken line located underneath of the polymorphic sequence. B Chromatin Immunoprecipitation (ChIP) assay was conducted in the BRM-positive cell line, TTC642 (wild type for Poly-1321/Poly-741) to determine whether HDAC9 and/or MEF2D can bind to the BRM promoter. No significant bindings of either MEF2D or HDAC9 were observed in TTC642 (p>0.05), compared to the IgG control. C ChIP was conducted in the BRM-negative Rhabdoid cell line, G401 (homo/homo for Poly-1321/Poly-741), to assess HDAC9 and/or MEF2D binding to the BRM promoter. Binding of both HDAC9 and MEF2D to the G401 promoter was observed at or near both the Poly 1321 and Poly 741 sites (p<0.05, compared to IgG control). D ChIP was conducted in the BRM-negative Rhabdoid cell line, KD (homo/wild type for Poly-1321/Poly-741), for HDAC9 and/or MEF2D binding to the BRM promoter. Binding of both HDAC9 and MEF2D to the BRM promoter at or near the Poly 1321 site was observed (p<0.05, compared to the IgG control), but no binding to the BRM promoter at or near the Poly 741 (wild type) site was observed (p>0.05, compared to the IgG control). E ChIP was conducted in the BRM-negative Rhabdoid cell line, KPMRT-NS (wild type/hetero for Poly-1321/Poly-741), for the binding of HDAC9 and/or MEF2D to the BRM promoter. Binding of both HDAC9 and MEF2D to the BRM promoter at or near the Poly-741 (hetero) site (p<0.05, compared to IgG control) was observed, but no binding of either HDAC9 or MEF2D to the BRM promoter at or near the Poly 1321 (wild type) site (p>0.05, compared to IgG control) was observed. F illustrates the BRM promoter reporter construct. This construct consisted of 10kb upstream of the BRM promoter as well as 10kb downstream of the translational start site. The luciferase gene linked was to the IRES-neomycin gene at the beginning of the translational site, with or without the two BRM polymorphic sites present in the BRM promoter. G shows the results of the ChIP experiment using the BRM luciferase reporter construct, where the BRM promoter contains either the presence (G401P) or absence (G401WP) of the two BRM polymorphic sites. When the BRM polymorphic sites were present (G401P), HDAC9 and MEF2D were found to bind to the BRM promoter at or near each BRM polymorphic site, respectively, whereas in the absence of these two BRM polymorphic sites (G401WP), HDAC9 and MEF2D demonstrated a lack of specific binding; that is, the binding was comparable to nonspecific IgG, which was used as a control.

We previously identified two polymorphic sites within the BRM promoter, which are a duplicate repeat of “TTTTAA” and a triplicate repeat of “TATTTTT” at the position of -1321 and -741, respectively, upstream of the transcriptional start site in the BRM promoter (Figure 7A). In Caucasians, these two polymorphic sites have an independent and joint frequency of ~20% and 6%, respectively, and these sites are in Hardy Weinberg equilibrium [21]. After analysis of BRM-deficient and BRM-positive cancer cell lines as well as primary lung tumors, we observed that the presence of these polymorphic sites statistically correlates the with loss of BRM expression [21]. We also analyzed the frequency of these polymorphic sites in the BRM-deficient Rhabdoid cancer cell lines and found a statistical correlation (using the Fisher Exact test) between BRM loss and the presence of the BRM polymorphic site Poly-1321 (P=0.02), but not Poly-741 (p=0.75) [21]. Consistent with the hypothesis that the BRM polymorphisms facilitate BRM silencing, the only BRM-positive Rhabdoid cell line (TTC-642) lacks both BRM polymorphisms.

BRM is an anticancer gene whose loss of expression statistically correlates with the presence of these BRM polymorphisms [21]. In turn, these polymorphisms are statistically correlated with cancer risk and a worse clinical outcome in a number of adult cancer types [21, 22, 43, 44]. Moreover, we have previously determined that HDAC9 and MEF2D underlie the silencing of BRM, as the shRNA knockdown of either gene results in the induction of BRM [25]. Moreover, a comparison of these polymorphic sites with known transcriptional binding sites has revealed that these BRM polymorphic sites may be relatively similar to several AT-rich binding sites of certain transcription factors but are highly homologous to the known binding sites (>92%) of the MEF2 family of transcription factors [21]. As MEF2 transcription factors are known to recruit a class II HDAC (HDAC9) to specific gene promoters in order to silence the target genes [45], this suggests that MEF2D and HDAC9 may function in a similar manner to specifically regulate BRM.

To determine if MEF2D and HDAC9 can bind to the BRM promoter, we conducted chromatin immunoprecipitation (ChIP) experiments in multiple Rhabdoid cell lines with varying BRM polymorphism genotypes. We first analyzed the BRM-positive cell line TTC-642, which is wild type/ wild type for both BRM polymorphic sites, for the presence of MEF2D and HDAC9; we observed essentially no recruitment of these proteins to the BRM promoter region (Figure 7B). However, in ChIP experiments using the BRM-deficient Rhabdoid cell lines G401 and KD, we observed increased binding for both MEF2D and HDAC9 only when the polymorphisms were present (Figure 7C and 7D). Ideally, ChIP would be performed in a BRM-deficient cell line that is wild type at Poly-1321 but homozygous at Poly-741 for complete analysis. Instead, we analyzed the KPMRT-NS Rhabdoid cell line, which is wild type/hetero for the -1321 and -741 polymorphisms, respectively; for this cell line, our ChIP showed binding of MEF2D and HDAC9 to the -741 (hetero) but not the -1321 (wild type) site (Figure 7E). These data clearly indicate that MEF2D and HDAC9 likely bind the BRM promoter at the polymorphic sites, as we detected binding of MEF2D and HDAC9 by ChIP only when the polymorphic sites were present.

As conclusions from data generated from different cell lines can be impacted by inherent differences between those cell lines, we next sought to conduct ChIP experiments in the presence of these polymorphisms in a genetically equivalent cell line. We constructed a Rhabdoid cell line where MEF2D and HDAC9 binding could be analyzed and compared as a function of the presence and absence of these polymorphisms in an otherwise clonal cell line. To accomplish this, we introduced a BRM promoter reporter construct into the G401 Rhabdoid cell genome via homologous recombination. This construct consisted of 10 kb upstream of the BRM transcriptional start site as well as 10 kb downstream of the translational start site residing within exon 2, as illustrated in Figure 7F. In this construct, a luciferase gene linked to an IRES-neomycin gene was placed at the beginning of the translational start site. Hence, after homologous recombination and insertion of this BRM promoter reporter construct, the endogenous BRM gene was disrupted such that it was no longer expressed. Instead, the luciferase gene, now under the control of BRM promoter (with or without the two BRM polymorphic sites) could be expressed as a measure of BRM promoter activity.

For this experiment, we obtained a number of daughter cell lines derived from single cells by dilutional cloning both with and without these polymorphisms. Comparing the luciferase activity from the six clonal daughter cell lines (3 each) which either did or did not harbor the BRM polymorphisms, we observed a ~7-8-fold higher luciferase expression (p<0.05) from the daughter cell lines harboring the BRM promoter construct without the BRM polymorphisms as compared to the cell lines harboring the BRM construct that included these polymorphisms (Supplementary Figure 6). This finding indicates that the presence of these BRM polymorphisms decreases the level of BRM expression, and thus they appear to have a functional role in BRM expression. Next we conducted ChIP experiments on two molecularly altered G401 Rhabdoid cell lines where one cell line harbored the BRM polymorphism (G401P) and the other did not (G401WP). We observed MEF2D and HDAC9 binding when the BRM polymorphisms were present (G401P cells); in comparison, we observed little to no binding of HDAC9 and MEF2D when the polymorphisms were absent (G401WP cells) (Figure 7G). When we repeated these experiments with additional clonal daughter cell lines, we observed greater binding only when the BRM polymorphisms were present. There was a 3-6-fold difference in the ChIP binding values between G401P and G401WP for both MEF2D and HDAC9. These data demonstrate that HDAC9 and MEF2D binding occurs within the BRM promoter at or near these polymorphic sites, as we observed that HDAC9 and MEF2D binding only occur when these polymorphic sites are present.

## DISCUSSION

While BAF47 clearly has an important role in Rhabdoid tumorigenesis, the finding that BRM is lost in addition to BAF47 adds another dimension to the evolution of our understanding of this tumor. BRM loss is thought to be an early event in the onset of cancer, since BRM polymorphisms have been found to be predictors of cancer risk. In turn, since BRM polymorphisms are functionally linked to BRM silencing, BRM loss could be an event which triggers the onset of cancer development. Our findings suggest that the silencing of BRM might occur before BAF47 loss, because BAF47 re-expression only causes a smaller increase in BRM mRNA expression (5-fold) in comparison to HDAC9 knockdown which drives the induction of BRM mRNA (12-14-fol---d). Alternatively, BRM loss and BAF47 loss may occur at the same time, since BAF47 can partially regulate BRM expression. Nevertheless, the epigenetic changes to BRM, as well as to p16 and EZH2, are consistent with the low mutation rate observed in Rhabdoid tumors [8]. More work is required to determine how BAF47 and BRM loss jointly contribute to the development of Rhabdoid tumor. In the present work, we have shown that not only does BRM loss occur in Rhabdoid tumors, but also that BAF47 regulates BRM, although the precise mechanism by which this occurs is not yet known. Since knockdown of BAF47 in another lung cancer cell line failed to change BRM expression levels, this mechanism is likely restricted to Rhabdoid tumors—or is dependent on other factors. Further, while it is known that th-ere is stoichiometric regulation among SWI/SNF subunits, where excess subunits are degraded and the loss of certain subunits (e.g. BAF155) precipitates the loss of other subunits (e.g. BAF60A, BRG1 and BAF47) [46], our data demonstrate that this mechanism of regulation does not occur between BAF47 and BRM.

The data we have presented demonstrate that the mechanism of BRM suppression in Rhabdoid tumors closely parallels that seen in lung cancer cell lines. Like lung cancer, in these Rhabdoid studies, we found that HDAC3, HDAC9, GATA3 and MEF2D regulate BRM. Moreover, HDAC9 was significantly overexpressed in all BRM-deficient Rhabdoid cell lines that were tested and in 5/5 Rhabdoid primary tumors, but neither HDAC3 nor MEF2D were overexpressed. Similar to lung cancer cell lines, GATA3 and MEF2D regulate both HDAC9 and BRM [25]. In both lung cancer and Rhabdoid cell lines, we have found that HDAC9 and MEF2D bind to the BRM promoter. Together, these findings suggest that the mechanism of BRM silencing is conserved. We also showed that the presence of at least the -1321 polymorphism correlates with BRM loss in Rhabdoid tumors. As these BRM polymorphisms are germline, the development of Rhabdoid tumors may be genetically linked, and the occurrence of Rhabdoid tumors may be partially predicted by the presence of these polymorphisms. This idea is supported by the fact that these BRM polymorphisms are known to be predictive of the development of lung, head/neck, and hepatocellular cancers thus far [21, 22, 47]. Future case control studies may reveal the relationships of these polymorphisms with cancer risk in other tumor types. However, the establishment of the risk of developing Rhabdoid sarcoma based on the presence of these polymorphisms would be difficult since the incidence of Rhabdoid tumors is relatively low.

The presence of these BRM polymorphisms and the loss of BRM expression have been linked to worse clinical outcomes in several adult tumor types [2, 44, 48]. Based these studies, one may wonder if BRM loss or the presence of these BRM polymorphisms or both similarly impact the response of Rhabdoid tumors to therapy. BRM loss and the presence of these polymorphisms might not be causative, but rather, might occur as the byproduct of a process such as the cancer-driven loss of heterozygosity (LOH). Indeed, the BRM locus is an area of LOH, and the single allelic loss in this region occurs in 40-60% of most solid cancers [49, 50]. There are two forms of Rhabdoid cancer, sporadic and familial, and it would be interesting to know if BRM loss occurs similarly or differently in the two forms and whether it contributes to the worse outcomes observed in patients with the familial form. Exactly how the BRM polymorphisms and BRM silencing indicate worse outcomes or more aggressive tumor types is not known. However, BRM re-expression microarray experiments show that BRM loss causes the down-regulation of a plethora of cell adhesion receptors, such as E-cadherin, CD44, and Ceacam1 integrin (data not shown), as well as the disruption of the function of tumor suppressors such as Rb and p53. Our ChIP experiments were based on PCR of a 300bp region; therefore, the area of MEF2D and HDAC9 binding cannot be precisely pinpointed from these experiments alone. As ChIP binding for MEF2D and HDAC9 was observed only in cell lines with these polymorphisms and not those that lacked the polymorphisms, MEF2D and HDAC9 were indirectly tied to the these polymorphic sites. Further underscoring the role of HDAC9 binding to these polymorphic sites, we conducted ChIP experiments on MEF2 and HDAC9 in Rhabdoid cell lines where we swapped in a BRM promoter that either contained or lacked the BRM polymorphisms. In this way, we could compare ChIP results in the same cell lines. Using this BRM promoter swap technology, we observed by ChIP that MEF2D and HDAC9 specifically bound to the BRM promoter only when these polymorphic sites were present. While these experiments are not quantitative, we observed about 3-5- fold higher binding of MEF2D to the promoter in the presence of these polymorphisms. This is not surprising, as these polymorphic sites are highly homologous to defined MEF2 binding sites.

The ultimate clinical goals of this research are to understand how Rhabdoid tumors avert growth control at the molecular level and to develop new avenues for therapy. Compounds such as LEE011, a CDK4/6 inhibitor that is currently in clinical trials [51], might not be effective, given that they do not restore the expression of BRG1 and BRM, which are required to facilitate Rb function as well as p130 and p107 [34]. Similarly, the pan-HDAC inhibitor vorinostat (SAHA) [52], which is currently being used against Rhabdoid tumors in clinical trials, would be expected to robustly induce BRM expression; however, since this compound also inhibits HDAC2, it would also result in BRM acetylation and inactivation. Given BRM's role in numerous pathways and its cooperation with key anticancer proteins such as Rb and p53, inactivation of BRM might thwart the activity of this drug in Rhabdoid tumors. Based on our experimental data, targeting HDAC9 might be an avenue of therapy for Rhabdoid tumors, since BRM re-expression seems to be important to block the growth of this tumor. If HDAC9 was therapeutically inhibited, it has two properties that make it an ideal clinical target. First, as HDAC9 has a tissue-restricted pattern of expression, its inactivation is less likely to create off-target effects and is therefore likely to be less toxic. Second, it is highly overexpressed in tumors, which indicates that such tumors are likely dependent on the expression of this gene; thus, even modest inhibition of HDAC9 could prove beneficial. To this end, Flavopiridol and other flavonoids may represent another viable therapeutic strategy, as they induce BRM by indirectly down-regulating HDAC9 as well as by inducing hypophosphorylated Rb, which are prerequisites for growth inhibition. Thus, understanding the epigenetic mechanisms of how BRM and other proteins are silenced can guide the intelligent use of targeted therapy.

## MATERIALS AND METHODS

### Cell Culture

Cell lines were grown in RPMI media supplemented with 5% fetal bovine serum, 1% Glutamax and 1% pen/strep. The G401 cell line was obtained from American Type Culture Collection (ATCC, Manassas, VA, USA). The BT12 and BT16 cell lines were obtained from Dr. P. Houghton (St. Jude Children's Research Hospital, Memphis, TN, USA). TM87, TTC642 and TTC1240 were obtained from Dr. T. Triche (Children's Hospital, Los Angeles, CA, USA). The KD and LM cell lines were obtained Dr. R. Handgretinger (Tübingen, Germany). KP-MRT-AN, KP-MRT-NS and KP-MRT-YML were obtained from Dr. H. Hosoi (Kyoto, Japan). For treatment with flavonoids, cells were plated in six-well plates or T75 flasks at about 60% density and were treated with the appropriate flavonoids. Flavonoids used for the analysis were purchased from Indofine (Hillsborough, NJ, USA) and Selleck Chemical (Houston, TX, USA). For RNA, cells were collected after 48h, whereas for protein, cells were collected after 72h. For transfection assays, cells were plated in T75 flasks at ~65% density and transfected with the plasmid of interest as previously described [25].

### Growth Inhibition Assay

Cell lines were plated in 6-well plates at a starting density of ~15%, and growth assays were conducted as previously described [17, 25]. Each test data point in the growth inhibition assay was normalized against the control data point obtained from cells transfected with the empty vector. Each data value is divided by the corresponding value of the empty vector control to generate a percentage value of growth inhibition.

### Quantitative PCR (qPCR)

Cells were lysed using Trizol reagent followed by extraction of total mRNA with an RNA extraction kit (Sigma-Aldrich, St Louis, MO). Complementary DNAs (cDNAs) were generated as previously described [25]. Primers used for the analysis are, BRM - 5'BRM-GATTGTAGAAGACATCCATTGTGG, 3'BRM-GACATATAACCTTGGCTGTGTTGA, and HDAC9 – 5'HDAC9- GAGCCACTTGCAGGACTGAG, 3'HDAC9 - GCTGCTTCTGGATTTGTTGC. All reactions were performed using SYBR Green/ROX qPCR Master Mix (SA Biosciences/Sigma-Aldrich). Fold differences in mRNA expression were calculated with the following formula,

2^(ΔCTtest - ΔCTcontrol)^ = fold difference

### Western Blotting

Following the treatment with flavonoids or the transfection experiments, cells were harvested and total protein was extracted using a urea-based lysis buffer as described previously [17, 25]. A rabbit polyclonal anti-BRM antibody was used for the detection of BRM at a dilution of 1:500 [17]. Mouse anti-phospho Rb (BD Biosciences, San Jose, California) was used at 1:250 for the detection of phsopho-Rb protein. Appropriate secondary antibodies (GE Healthcare, UK) were used at a dilution of 1:2000. GAPDH antibody (GeneTex Inc., Irvine, CA, USA) was used as the loading control.

### Generation of RNA Interference Knockdowns

All pLKO.1-shRNA were obtained from Open Biosystems. Each shRNA plasmid was introduced by transient transfection in 293T cells in combination with VSGS and psPAX2 plasmids to generate competent virus, which was harvested daily for five days and stored at 40x concentration by volume with RPMI media without FBS. Each cell line was incubated with the virus for 6 hours and then replaced with RPMI supplemented with 10% FBS; the whole process was repeated three times, after which the cells were selected in puromycin at 5-10μM for 1 week week. The knockdown of the respective targeted gene was confirmed by western blotting.

### Immunohistochemical Staining

Immunohistochemical staining was conducted as previously described [17, 25, 53]. Anti-BRM rabbit antibody was used at dilution of 1:500; rabbit polyclonal anti-HDAC9 antibody (Abcam, Cambridge, MA, USA) was used at a dilution of 1:50, and the rabbit polyclonal anti-BAF47 (Santa Cruz Biotechnology, Dallas, TX, USA). A goat anti-rabbit biotinylated (GE Healthcare, UK) secondary was used at a 1:200 dilution.

### Promoter Swap Study

BRM promoter reporter constructs with or without the BRM polymorphic sites (Poly-1321/Poly-741) for this study were custom designed by Spectra Genetics Inc. (Pittsburgh, PA, USA). TALEN recombinase was obtained from Cellectis Bioresearch (Cambridge, MA, USA). For the experiment, cells were plated at ~75% density in 24-well plates and transfected with plasmids harboring the constructs using Polyplus Jet Prime Reagent (VWR, Radnor, PA, USA). A total of 1 µg of TALEN recombinase was then added to the media. Subsequently, cells were selected with neomycin and plated at a very low density in 100 mm plates. Daughter cell lines were generated from single cells by dilutional cloning. The efficiency of the integration was determined by a luciferase assay with OneGlo reagent (Promega, Madison, WI, USA) and the FLx800 microplate reader (Biotek, Winooski, VT, USA).

### Chromatin Immunoprecipitation Assay (ChIP)

For Chromatin Immunoprecipitation (ChIP) assay, cells were cultured in T225 flasks and treated with either vehicle or 600nM of TSA for 24 hours. In the baseline state or the uninduced state in a BRM-negative cell line, the chromatin in and around the BRM locus remained closed; as such, no binding to the DNA occurs. To determine if a certain protein binds to the BRM locus, one must turn-on the BRM gene (induce BRM). In doing so, the BRM silencing mechanism is halted or prevented from closing the chromatin. In this open state, we can see which proteins bind to the BRM locus. The TSA induces BRM by inhibiting the de-acetylase activity of HDAC3 and HDAC9 but does not prevent their binding to the BRM promoter; TSA probably freezes the binding and prevents the completion of the BRM silencing mechanism. The cells were cross-linked using 1% formaldehyde for 10 min at room temperature, washed and lysed in lysis buffers (1% SDS, 10mM EDTA-pH 8, 50mM Tris-HCl-pH 8). The cross-linked DNA was sheared with Diagenode Bioruptor UCD-200 Sonicator (Denville, NJ, USA) for 12 minutes. The resulting cell extract was precleared with Protein G magnetic beads (GenScript, Piscataway, NJ, USA) at 4°C for 2 hours. A total of 4 µg of the appropriate antibody was added to each sample and incubated overnight at 4°C with gentle rotation. To capture antibody-protein-DNA complexes, 80 µL of protein G magnetic beads were added to each sample and incubated at 4 °C for 4 hours. Beads were washed and antibody-protein-DNA complexes were eluted from the beads. To reverse the cross-linking, the complex was incubated with 200 mM NaCl for 5 hours at 65°C, followed by the addition of Proteinase K and further incubation for 2 h at 45°C to digest the proteins. The DNA was then purified and quantified by qPCR using the Applied Biosystems® StepOne and StepOnePlus Real-Time PCR Systems (Life Technologies, Grand Island, NY, USA). For amplification of the polymorphism site -1321, the following primers were used: 5'BRMprom-6339D: AAGAATCCTCAACCAGATAGTCACA, 3'BRMprom-6507D: CAGGGGCCTATTATTTTAGACTCA. The primers for the amplification of polymorphism site- 741 were: 5'BRM prom- 6955: TTTGGAAGCTTGCAGTCCTT, 3'BRM prom-7089: CCGGCTGAAACTTTTTCTCC. For data analysis, each immunoprecipitation sample was compared to the standard curve generated by amplifying serial dilutions of its input.

### Statistical Analysis

Students t-test was used to compare the statistical significance of different treatment. The error bar represents the SEM of experiments performed in triplicate.

## SUPPLEMENTARY FIGURES AND TABLE


