# Supplementary material for: The silencing of the SWI/SNF subunit and anticancer gene BRM in Rhabdoid tumors
Source: Oncotarget. 2014 May 4;5(10):3316–32. doi: 10.18632/oncotarget.1945 (PMC4102812; doi:10.18632/oncotarget.1945)
Supplement: Supplementary file 1 [file oncotarget-05-3316-s001.pdf]

The silencing of the SWI/SNF subunit and anticancer gene sBRM in Rhabdoid tumors

Supplementary Material

Supplementary Table 1: Table showing the status of BRM polymorphisms (-741/-1321) and BAF47 in Rhabdoid cell lines used in the study. Column 1 listed the name of the 11 Rhabdoid cell lines. Column 2 and 3 listed the status of the two BRM polymorphisms, designated as Poly 741 and Poly 1321, in the BRM promoter. Column 4 shows the defects associated with BAF47 in these cell lines.

Cell Lines	BRM Polymorphism		BAF47
	Poly 741	Poly 1321	Defects
KPMRT-AN	WT	HETERO	Deleted
KPMRT-YML	HETERO	HETERO	No
BT-12	HETERO	HOMO	Deleted
1240	HETERO	HOMO	G646T (nonsense mutation)
KPMRT-NS	HETERO	WT	No
TM-87	HOMO	HETERO	Deleted
BT-16	HOMO	HOMO	Deleted
G401	HOMO	HOMO	Deleted
KD	WT	HOMO	Deleted (Exon 4 & 5)
LM	WT	HOMO	Deleted
TTC-642	WT	WT	C118T (nonsense mutation)

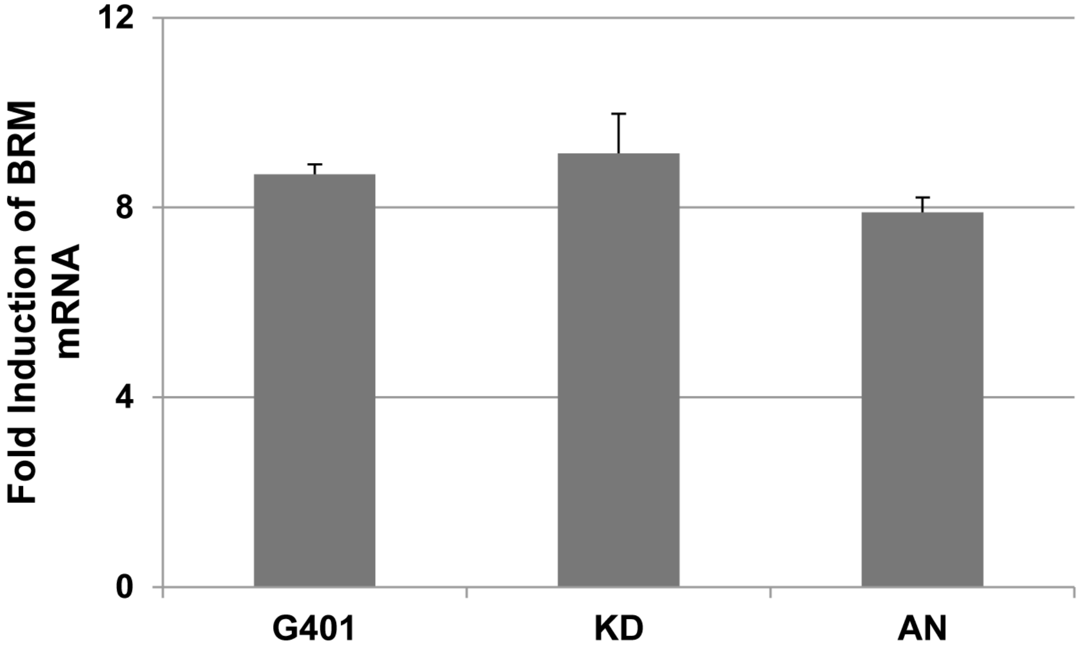

Supplementary Figure 1 demonstrates induction of BRM mRNA following the treatment of Rhabdoid cell llines, G401, KD and KPMRT-AN with 250nM of Flavopiridol for 48 hours.

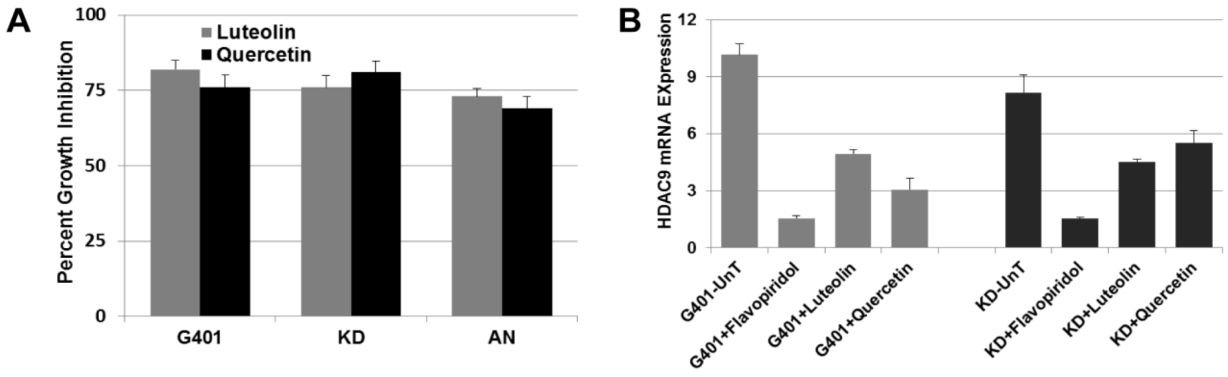

Supplementary Figure 2: Panel A demonstrates cellular growth inhibition (80-90%) following the treatment of Rhabdoid cell lines, G401, KD and KPMRT-AN with 3μM of indicated flavonoids over a period of 5 days.

Panel B illustrates the inhibition of HDAC9 mRNA ($p<0.05$) following the treatment of Rhabdoid cell lines, G401 and KD with 250nM of Flavopiridol, 3μM of Luteolin or 3μM of Quercetin. UnT represents the parental control cell lines.

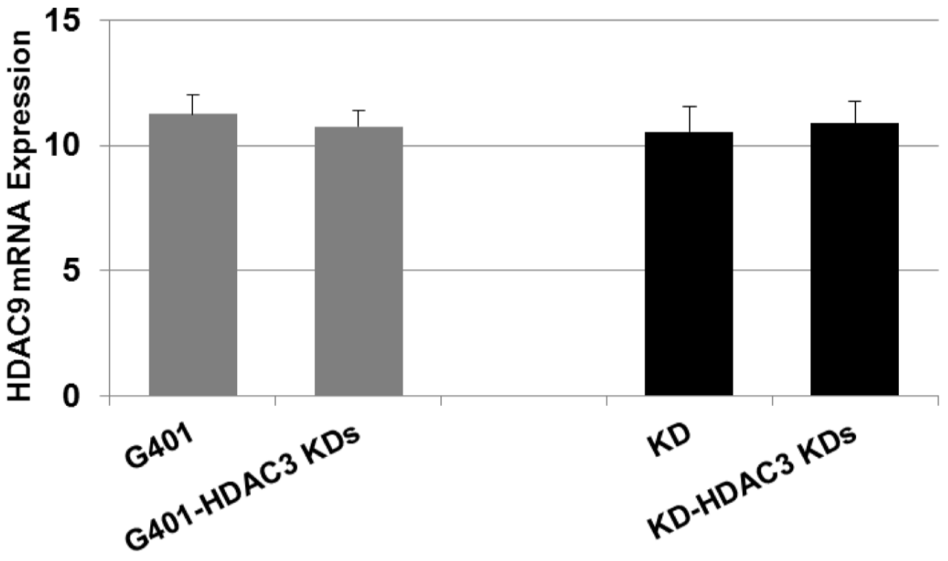

Supplementary Figure 3 demonstrates the level of HDAC9 mRNA level following the gene-specific shRNA mediated knockdown of HDAC3 in G401 and KD cell lines. No statistically significant changes in the HDAC9 level was observed following HDAC3 knockdown ($p>0.05$)

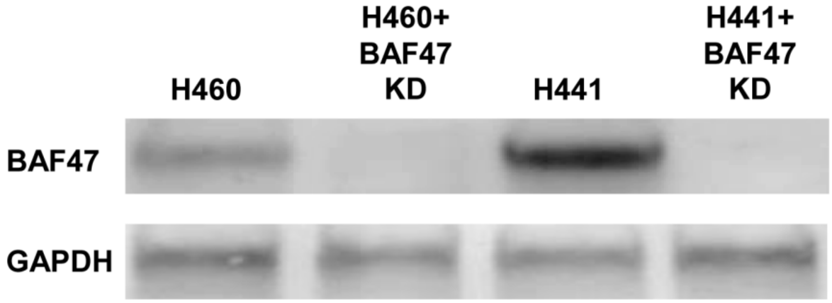

Supplementary Figure 4A: Western Blot showing the efficiency of BAF47 knockdown by gene-specific shRNA in H460 and H441 cell lines.

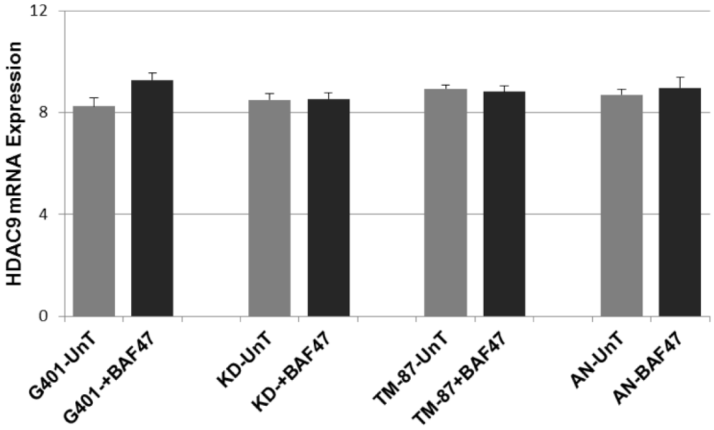

Supplementary Figure 4B: demonstrates the level of HDAC9 mRNA level following re-expression of BAF47in Rhabdoid cell lines, G401, KD, TM-87 and KPMRT-AN. No statistically significant changes in the HDAC9 level was observed following BAF47 expression ($p>0.05$)

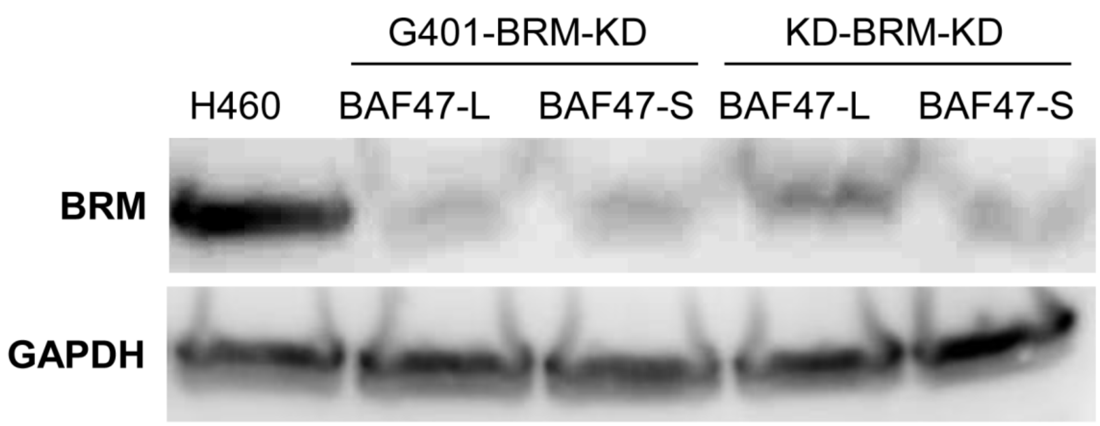

Supplementary Figure 5: Demonstrates the level of BRM protein after the transduction of long (BAF47-L)- and short (BAF47-S)-form of BAF47 in G401 and KD cell lines harboring the anti-BRM shRNA. No apparent differences in BRM expression was observed in either of the cell lines following the transduction of long- or short-form of BAF47.

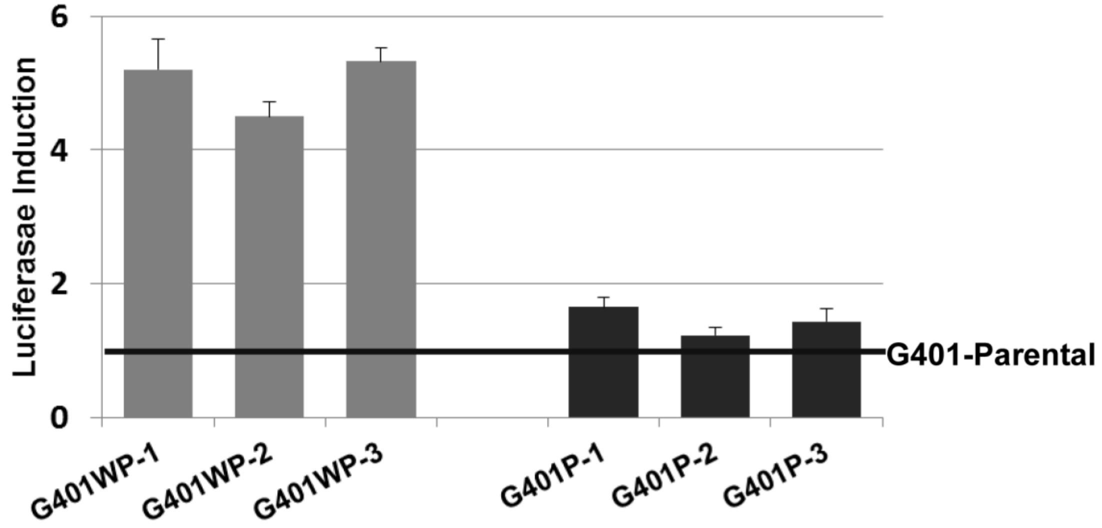

Supplementary Figure 6: Graph showing the luciferase output as a measure of BRM-promoter activity in molecularly altered G401 cell lines, G401WP (without BRM polymorphisms, -1321 and -741), and G401P (with BRM polymorphisms, -1321 and -741). The luciferase induction was normalized against G401 parental cell line (taken as 1). The daughter cell lines without the polymorphic sites (G401WP-1, 2 and 3) displayed significantly higher luciferase output ($p<0.05$) compared G401 cell lines harboring the BRM polymorphisms (G401P-1, 2 and 3).
